# In silico prediction of the action of bromelain on PI3K/Akt signalling pathway to arrest nasopharyngeal cancer oncogenesis by targeting phosphatidylinositol-4,5-bisphosphate 3-kinase catalytic subunit alpha protein

**DOI:** 10.1186/s13104-024-06995-2

**Published:** 2024-11-26

**Authors:** Alyaa Syafiqah Shamsuri, Edmund Ui -Hang Sim

**Affiliations:** Faculty of Resource Science and Technology, Universiti Malaysia Sarawak, Kota Samarahan, Sarawak, 94300 Malaysia

**Keywords:** Nasopharyngeal carcinoma, Molecular docking simulation, Protein-protein interactions, Bromelain, PIK3CA

## Abstract

**Objective:**

This research investigates the potential anti-tumour effects of bromelain, an aqueous extract from pineapple stems and fruits, on nasopharyngeal cancer (NPC). While bromelain is known for its medicinal properties in various cancers, its impact on NPC remains unexplored.

**Results:**

Using in silico methods, we studied the predicted interactions between bromelain and key proteins involved in NPC oncogenesis, specifically β-catenin, PIK3CA, mTOR, EGFR, and BCL2. Molecular docking strategies were performed using a myriad of computational tools. A 3D model of bromelain was constructed using SWISS-MODEL, followed by molecular docking simulations performed with ClusPro. The binding affinities of the docked complexes were evaluated using HawkDock, and the interactions were analysed with LigPlot+. The docking scores indicated potential spontaneous interactions, with binding affinities based on being − 103.89 kcal/mol (PIK3CA), -73.16 kcal/mol (EGFR), -71.18 kcal/mol (mTOR), -65.22 kcal/mol (β-catenin), and − 57.48 kcal/mol (BCL2). LigPlot + analysis revealed the presence of hydrogen bonds, hydrophobic interactions, and salt bridges, indicating stable predicted interactions.

**Conclusion:**

Our findings suggest that bromelain can target key proteins involved in NPC oncogenesis, with the strongest affinity towards PIK3CA. This suggests a hypothetical insight into bromelain’s anticancer effects on NPC through the modulation of the PI3K/Akt signaling pathway.

**Supplementary Information:**

The online version contains supplementary material available at 10.1186/s13104-024-06995-2.

## Introduction

Bromelain is a bioactive compound obtained from pineapple (*Ananas comosus*) aqueous extract that is widely studied as a drug candidate. It is known to have medicinal properties such as antimicrobial, antithrombotic and anti-inflammatory effects [1]. Previous in vitro and in vivo studies have revealed its anticancer effects on various types of cancer such as lung [2, 3], brain [4], colorectal [5, 6], skin [7], and breast [8–10] cancer. However, to date, literature evidence of this effect on Nasopharyngeal carcinoma (NPC) is lacking, including those from in silico findings.

NPC is a type of head and neck squamous cell cancer (HNSCC) that is preferentially common in Asia, particularly Southeast Asia [11, 12]. NPC pathogenesis involves several crucial signalling pathways including those that regulate cell proliferation, metastasis and apoptosis inhibition [13]. Several key proteins in these pathways can be targeted for in silico studies in order hypothetical insights into the anticancer potentials of bromelain. The essential NPC-associated signalling pathways include the EGFR, phosphatidylinositol 3‑kinase (PI3K)/protein kinase B (Akt), and Wnt/β-catenin pathways. Dysregulation of the EGFR pathway is linked to disease recurrence, tumour migration, as well as poor prognosis and lower survival rates in NPC patients [14–16]. Abnormal regulation of the PI3K/Akt/mammalian target of rapamycin mTOR signalling pathway is associated with proliferation, migration and invasion in NPC cells [17]. Radioresistance in NPC is associated with dysregulation of the Wnt/β-catenin pathway [18, 19] [18, 19]. In addition, the apoptosis pathway plays a crucial role in NPC tumorigenesis, during which the silencing of proapoptotic factors by antiapoptotic factors such as BCL2 can lead to tumour progression [20]. Taken together, these findings indicate that the target receptor proteins (key proteins crucial for the regulation of the aforementioned pathways) chosen for our study were EGFR (for the EGFR pathway), PIK3CA and mTOR (for the PI3K/Akt/mTOR pathway), β-catenin (for the Wnt/β-catenin pathway), and Bcl2 (for the apoptosis pathway).

The identification of interactions between drugs and targets is a critical prerequisite steps in drug development [21]. Drug-target interaction (DTI) prediction via a computational approach is a common contemporary strategy that is empirically reliable, time-saving, and cost effective [22]. One of the computational methods used to simulate DTI at the molecular level is the molecular docking approach. In this study, this in silico method was carried out to determine the feasibility and characteristics of simulated interactions between bromelain and the key proteins associated with NPC oncogenesis.

## Methods

### Preparation of the Bromelain structure

A 3D model of stem bromelain was generated using the SWISS-MODEL workspace server due to the significant heterogeneity observed in the available experimental crystal structure (PDB ID: 6YCE) and the need for a consistent and well-characterized sequence for our study. The model was generated based on homology modelling [23, 24], using the input sequence of 212 amino acids from the UniProt KB database entry P14518 (BROM2_ANACO). The template was chosen as it closely resembles target sequence. The model’s quality was assessed using QMEAN score, Ramachandran Plot, ERRAT, and VERIFY3D via the SAVES server. PyMOL software was used to generate the root mean square deviation (RMSD) through superimposition, and energy minimization was performed using Swiss-PdbViewer [25].

### Preparation of target receptors

Five target receptor proteins of bromelain (β-catenin, PIK3CA, mTOR, EGFR, and BCL2) were selected. The 3D crystallographic structures of these proteins were obtained from the RCSB PDB website (PDB IDs: 7AFW, 5DXT, 4DRI, 3POZ, 4LVT). Preparation involved removing ligands, water molecules, and side chains using Discovery Studio 2021. Polar hydrogen and Kollman charges were added using AutoDock Tools [26].

### Molecular docking and analysis

Molecular docking was performed using the ClusPro server, which involves three steps: rigid body docking with PIPER, clustering of the top 1000 structures using RMSD, and structural refinement via energy minimization [27–31]. The balanced category was chosen for the docked model, with scoring based on the formula:

$${\rm{E = 0}}{\rm{.40}}{{\rm{E}}_{{\rm{rep}}}}{\rm{ + - 0}}{\rm{.40}}{{\rm{E}}_{{\rm{att}}}}{\rm{ + 600}}{{\rm{E}}_{{\rm{elec}}}}{\rm{ + 1}}{\rm{.00}}{{\rm{E}}_{{\rm{DARS}}}}$$

The ligand and receptors used were bromelain and the target proteins, respectively. The best solution with the highest cluster size and lowest free energy was selected.

The binding affinities were predicted using the HawkDock server [32], which calculates the binding free energy (BFE) using the MM-GBSA approach [33]. BFEs were estimated based on Van der Waals forces, electrostatic bonds, Generalized Born model polar solvation free energies, and empirical model non-polar solvation free energies. LigPlot + software analysed interacting residues, assessing hydrogen bonds, hydrophobic residues, and salt bridges [34, 35]. The strength of the hydrogen bonds was evaluated by donor-acceptor distance, ranging from 2.2 Å to 4.0 Å.

## Results

### 3D model structure of bromelain and quality assessment

The SWISS-MODEL homology-based modelling protocol required an initial target-template sequence comparison before constructing the computational structure. Sequence alignment between bromelain and the template protein (PDB ID: 6YCE) revealed a 95.73% sequence homology. Based on this template, a 3D structural model of bromelain was constructed and evaluated. The QMEAN score of -0.15 indicated the model’s acceptability, as scores around zero, not deviating more than 1 standard deviation from the mean, suggest high-quality models [25]. The Ramachandran plot showed 89.1% of residues in the favoured region with no outliers, affirming stereochemical quality. The low RMSD score of 0.079Å indicated high similarity with the template. High ERRAT (96.4) and VERIFY3D (100%) scores further confirmed the model’s quality. Collectively, these assessments validated the constructed model for use in molecular docking assays.

### Molecular docking and assessment

PyMOL illustrations of the docked complexes for EGFR-bromelain, mTOR-bromelain, BCL2-bromelain, PIK3CA-bromelain, and β-catenin-bromelain are shown below. The MM-GBSA total binding free energy (BFE) from HawkDock showed that PIK3CA had the highest affinity for bromelain, followed by EGFR, mTOR, β-catenin, and BCL2 (Table 1). All docked complexes had negative values, indicating potential spontaneous reactions. The more negative the value, the greater the binding affinity. LigPlot + analysis revealed hydrogen bonds, hydrophobic residues, and salt bridges in all complexes (Table 2). The interacting residues within the docked complexes are shown for EGFR-bromelain, mTOR-bromelain, BCL2-bromelain, PIK3CA-bromelain, and β-catenin-bromelain. Additional figures viewed from PyMOL shows the illustration of 3D model structure of bromelain and the illustration of docked complex of each proteins with bromelain respectively; Ligplot + illustrations of docked complexes shows the interacting residues in more detail [see Supplementary Material 1].

**Table 1:** Values of docking scores, MM/GBSA, Van Der Waal potentials (VDW), electrostatic potentials (ELE), polar solvation free energies (from Generalised Born (GB) model prediction), and nonpolar contribution to the solvation (SA) free energies (from empirical model calculation) of the docked complexes

Receptor	Cluster size	ClusPro Score		MM-GBSA total BFE (kcal/mol)	VDW	ELE	GB (kcal/mol)	SA (kcal/mol)
		Centre	Lowest energy (KJ/mol)					
EGFR	74	-603.1	-718.6	-73.16	-79.25	-365.64	383.45	-11.72
mTOR	142	-697.0	-768.9	-71.18	-82.52	-595.35	618.22	-11.53
BCL2	104	-630.0	-953.5	-57.48	-95.19	-482.25	531.37	-11.40
PIK3CA	129	-781.0	-839.7	-103.89	-125.29	-812.14	851.35	-17.81
β-catenin	127	-569.3	-699.8	-65.22	-84.40	-37.12	68.70	-12.40

**Table 2:** The hydrogen bonds, hydrophobic residues and salt bridges of the docked complexes obtained from dimplot results of LigPlot + analysis

Target receptor	H-bonds			Hydrophobic residues		Salt bridges	
	Receptor residues	Bromelain residues	Distance (Å)	Receptor residues	Bromelain residues	Receptor residues	Bromelain residues
EGFR	Glu928 Asp954 Asp954 His888 Ala955 Asp956 Ser925 Ser921 Arg889 Arg889 Lys867	Arg112 Gly60 Arg70 Asn157 Tyr61 Lys64 Arg115 Arg115 Ser155 Asp135 Asn137	2.87 2.98 2.70, 2.86 2.94 2.88 2.51, 2.53 2.90 2.74, 3.03 2.71, 3.23 2.75 2.93	Leu883 Glu884 Leu887 Ile890 Ile953	Lys59 Gly66 Trp67 Phe69 Leu156 Asp204	Glu928 Glu922	Arg112 Arg115
mTOR	Trp2101 Phe2039 Glu2032 Glu2032 Tyr2074 His2106 His2106 Tyr2105 Tyr2105 Arg2109 Arg2109 Arg2109 Arg2110	Tyr61 Lys64 Lys59 Arg70 Arg115 Thr154 Leu156 Trp67 Glu68 Glu68 Arg112 Asp204 Ile203	3.21 2.64 2.74 2.77, 2.73 2.77 3.07 3.21 3.00 3.09 2.72 2.67 2.79 2.95	Ser2035 Asp2102 Phe2108 Ser2112	Gly66 Phe69 Ala133 Asn157 Leu206	Arg2109 Arg2110	Glu68 Asp204
BCL2	Thr129 Glu132 Arg124 Asn169 Asn169 Pro165 Glu162	Arg115 Arg115 Tyr110 Arg70 Tyr61 Tyr61 Lys64	3.09 2.66 2.80 2.76, 2.77 2.81 2.67 2.60	Pro120 Phe121 Thr122 Ala123 Gly125 Ala128 Leu166 Leu172 Trp173	Gly24 Cys26 Trp27 Gly65 Gly66 Trp67 Phe69 Ser155 Leu156 Asn157 Ile203 Asp204	Glu162	Lys64
PIK3CA	His510 Ser507 Arg557 Trp328 Asn331 Asn331 Asn331 Glu494 Lys325 Lys325 Ser482 Trp479 Arg335 Ser481 Asp390 Asp395 Asp578 Arg502 Arg502 Arg502	Thr98 Asp99 Tyr61 Trp67 Glu68 Asp204 Arg112 Lys64 Leu156 Ser155 Ser155 Arg115 Arg115 Thr154 Tyr110 Arg70 Arg70 Leu55 Asp56 Ala58	2.80 2.91 2.67 2.71 2.95 2.86 2.61, 2.79 2.51 2.48 2.76, 2.52 2.96 2.72, 2.78 2.67 2.78 2.87 2.72 2.68 3.27 2.71, 2.84 2.61, 2.71	Ser332 Tyr392 Phe480 His495 Trp498 Ser509 Leu513	Cys57 Lys59 Gly60 Gly62 Phe69 Ser77 Lys79 Lys93 Cys96 Leu206	Asp478 Asp395	Arg115 Arg70
β-catenin	Lys233 His224 Arg185 Glu226 Asn261 Leu264 His265 Glu267 Arg225 Arg225 His186 Arg190 Arg190	Asn116 Tyr61 Tyr61 Arg115 Arg70 Tyr110 Arg112 Arg112 Glu68 Asp204 Leu156 Leu156 Ser155	2.58 2.94 2.61 2.69 2.60 2.84 2.92 3.10 2.72 2.82 3.25 2.71 2.66, 2.78	His223 Leu228 Leu229	Trp67 Phe69 Ala111 Ala133 Ala159 Ile203	Glu182 Arg225	Lys64 Glu68

According to LigPlot + results, the bromelain-PIK3CA interaction involved the highest number of hydrogen bonds [25], followed by EGFR [15], mTOR and β-catenin (14 each), and BCL2 [8]. In the bromelain-PIK3CA complex, 15 and 5 interacting residues formed single and double hydrogen bonds, respectively. The shortest distance (2.48 Å) between two interacting residues in a hydrogen bond was found in the bromelain-PIK3CA complex involving Lys325 (PIK3CA) and Leu156 (bromelain). For hydrogen bonds exceeding 3.00 Å, the bromelain-mTOR complex had the most (35.7%), and the bromelain-PIK3CA complex had the least (4%). Comparable numbers of longer hydrogen bonds were found in bromelain-EGFR, bromelain-BCL2, and bromelain-β-catenin complexes, with 13.3%, 12.5%, and 14.3%, respectively. The hydrogen bond quantity and distances partly explain the higher binding affinity of bromelain with PIK3CA, as shown by the MM-GBSA total BFE results. Despite having the fewest hydrogen bonds, the bromelain-BCL2 complex had the highest number of hydrophobic interactions (21 residues), with nine from BCL2 and twelve from bromelain. The bromelain-PIK3CA interaction involved 17 hydrophobic residues, the second largest number after BCL2. Salt bridge interactions were comparable (two each) among all complexes except for the bromelain-BCL2 complex, which had one (Glu162-Lys64).

The BFE contribution by the top five residues for receptor proteins and bromelain based on HawkDock analysis (Table 3) was consistent in showing PIK3CA as the most preferred target receptor for bromelain. The bromelain-PIK3CA complex had the lowest average BFE for the top five receptor (-6.188 kcal/mol) and bromelain (-6.226 kcal/mol) residues compared to other complexes. Lower BFE values indicate higher binding affinity. For other complexes, the average BFE for the top five residues were: -4.362 kcal/mol for receptor and − 5.554 kcal/mol for bromelain in bromelain-EGFR; -6.096 kcal/mol for receptor and − 5.792 kcal/mol for bromelain in bromelain-mTOR; -4.134 kcal/mol for receptor and − 3.818 kcal/mol for bromelain in bromelain-BCL2; and − 5.526 kcal/mol for receptor and − 4.298 kcal/mol for bromelain in bromelain-β-catenin.

**Table 3:** Top 5 residues of receptor and ligand (bromelain) which contribute to the BFE of the docked complexes based on HawkDock analysis

Target receptor	Receptor residues	BFE contributed (kcal/mol)	Bromelain residues	BFE contributed (kcal/mol)
EGFR	Arg889 His888 Leu887 Asp954 Ile953	-8.66 -4.11 -3.26 -2.97 -2.81	Arg112 Arg70 Trp67 Arg115 Tyr61	-7.46 -6.23 -5.27 -4.88 -3.93
mTOR	Arg2109 Tyr2105 Glu2032 Phe2039 His2106	-10.5 -7.26 -5.30 -4.16 -3.26	Asp204 Glu68 Arg70 Tyr61 Trp67	-8.14 -6.10 -5.08 -4.87 -4.77
BCL2	Phe121 Asn169 Arg124 Glu162 Thr122	-6.51 -5.60 -3.37 -2.74 -2.45	Tyr61 Trp67 Phe69 Lys64 Leu156	-5.30 -4.48 -4.13 -3.22 -1.96
PIK3CA	Asn331 Asp395 Asp578 Trp498 Tyr392	-8.64 -6.87 -5.67 -5.34 -4.42	Arg70 Trp67 Arg115 Leu156 Tyr61	-11.78 -5.82 -5.30 -4.86 -3.37
β-catenin	Arg225 His265 Glu226 Leu229 His224	-13.60 -6.71 -2.63 -2.40 -2.29	Glu68 Trp67 Phe69 Arg112 Tyr61	-6.81 -4.44 -4.19 -3.10 -2.95

## Discussion

Our in silico analysis suggests that bromelain interacts with key proteins (PIK3CA, EGFR, mTOR, β-catenin, and BCL2) from signalling pathways associated with NPC tumorigenesis. Notably, bromelain-PIK3CA interaction is the strongest, exhibiting the highest number of H-bonds, and strongest binding affinity.

The PI3K/Akt/mTOR pathway is involved in various biological functions including cell differentiation, proliferation, survival, as well as cell growth [36]. The dysregulation of this pathway has been strongly implicated in the development and progression of multiple cancers, including NPC. PIK3CA, also known as the p110α protein, is the catalytic subunit of phosphatidylinositol 3-kinase (PI3K), which plays a crucial role in this pathway. Mutation of *PIK3CA* usually occur in exons 9 (helical domain and 20 (kinase domain) and it has been reported in several types of cancer including head and neck cancer [37, 38]. *PIK3CA* mutations have also been shown to be oncogenic by promoting the growth of cancer cells as well as invasion [39, 40]. Evidence of *PIK3CA* gene mutations involving the previously mentioned exons 9 and 20 has been shown before in 9.6% of NPC cases (*n* = 73) [41]. Mutation of the *PIK3CA* gene reportedly is also associated with NPC where its elevated expression has been linked to advanced stages in NPC [42]. Other than that, *PIK3CA* amplification as well as overexpression of its gene product, p110α has also been reported [43]. This may lead to the activation of the downstream cascades of the PI3K pathway. In fact, increased in PIK3CA is associated with poor prognosis of NPC patients [44]. Given the critical role of PIK3CA in NPC, the interaction between bromelain and PIK3CA observed in our study could suggest a mechanism by which bromelain may exert its anticancer effects. Specifically, the strong binding affinity observed in our in silico analysis, characterized by multiple hydrogen bonds and favourable binding energies, hints at a possible inhibitory interaction that may reduce PIK3CA activity or expression in NPC.

The effects of bromelain on PI3K/Akt pathway has been studied before whereby through western blot analysis, it was found that bromelain significantly reduced the expression of PI3K in the carcinogenesis of colorectal cancer [6]. This aligns with our findings, suggesting that bromelain’s interaction with PIK3CA could similarly inhibit this pathway in NPC, potentially leading to decreased tumorigenesis. This information, together with the potential interaction between PIK3CA with bromelain from our result may suggest that bromelain could potentially inhibit the expression of PIK3CA in NPC.

Other proteins also merit attention. EGFR activation triggers signalling cascades, including the PI3K/Akt pathway [45], and its overexpression is common in many cancers including NPC, and is linked with tumour recurrence, migration, and poor prognosis in NPC [14–16, 46]. The interaction between bromelain and EGFR observed in our study could indicate that bromelain might interfere with EGFR-mediated activation of the PI3K/Akt pathway, thereby potentially reducing NPC progression. mTOR, part of the PI3K/Akt/mTOR pathway, is associated with poor prognosis in late-stage NPC [47, 48]. Bromelain’s interaction with mTOR may suggest a similar inhibitory effect, potentially disrupting downstream signaling necessary for NPC tumorigenesis. In addition, the overexpression of BCL2, a key player in the apoptosis pathway, in NPC cells has been established since the 90s and has been implicated in NPC’s early stages due to its abnormal expression inhibiting apoptotic activities [49, 50]. Bromelain’s potential interaction with BCL2 might restore apoptotic processes in NPC cells, thus reducing their viability. β-catenin, a crucial member of the Wnt/β-catenin pathway, is associated with poor prognosis in NPC due to its abnormal expression patterns [51, 52] and the interaction of bromelain with β-catenin could suggest that bromelain interferes with the Wnt/β-catenin pathway.

Our findings provide a preliminary mechanistic insight into how bromelain may interact with and potentially modulate the activity of key proteins involved in NPC tumorigenesis. Further studies, including molecular dynamic simulations and experimental assays, are needed to validate bromelain’s anticancer effects on NPC, particularly its interaction with PIK3CA.

## Conclusion

In silico data indicate that bromelain interacts with key proteins; PIK3CA, EGFR, mTOR, β-catenin, and BCL2 involved in NPC tumorigenesis, with the strongest binding affinity observed with PIK3CA. These findings suggest a potential mechanism by which bromelain may exert anticancer effects on NPC, particularly through modulation of the PI3K/Akt pathway. However, it is important to note that these results are based on computational predictions and remain hypothetical. Further experimental validation is essential to confirm these interactions and their biological relevance in NPC.

### Limitations

The limitation of this study is that it relies solely on in silico methods, which may not fully capture the complexities of in vivo environments. The predictions based on molecular docking and binding affinity calculations require experimental validation through in vitro and in vivo assays to confirm bromelain’s anticancer potential as previously mentioned.

## Electronic supplementary material

Below is the link to the electronic supplementary material.

Supplementary Material 1

## Data Availability

All data generated or analysed during this study are included in this published article and its additional files.

## Abbreviations

Akt: Protein kinase B
BCL2: B-cell lymphoma 2
BFE: Binding free energy
DTI: Drug-target interaction
EGFR: Epidermal growth factor receptor
ELE: Electrostatic
FFT: Fast Fourier transform
GB: Generalised born model polar solvation free energies
H-bond: Hydrogen bond
HNSCC: Head and neck squamous cell cancer
mTOR: Mammalian target of rapamycin
MM-GBSA: Molecular mechanics-generalised born surface area
NPC: Nasopharyngeal cancer
PDB: Protein databank
PI3K: Phosphatidylinositol 3‑kinase
PIK3CA: Phosphatidylinositol-4,5-bisphosphate 3-kinase catalytic subunit alpha
QMEAN: Qualitative model energy analysis
RCSB PDB: Research Collaboratory for Structural Bioinformatics Protein Drug Bank
RMSD: Root mean square deviation
SA: Empirical model non-polar solvation free energies
VDW: Van der Waal
