# Supplementary material for: In silico prediction of the action of bromelain on PI3K/Akt signalling pathway to arrest nasopharyngeal cancer oncogenesis by targeting phosphatidylinositol-4,5-bisphosphate 3-kinase catalytic subunit alpha protein
Source: BMC Res Notes. 2024 Nov 26;17:346. doi: 10.1186/s13104-024-06995-2 (PMC11600585; doi:10.1186/s13104-024-06995-2)
Supplement: Supplementary file 1 — Supplementary Material 1 [file 13104_2024_6995_MOESM1_ESM.pdf]

Additional file 1

This file contains figures and illustrations that shows more details to the results obtained from this study. Structures of 3D bromelain model, docked complexes of each key proteins with bromelain and interacting residues from the docked complexes are included in this file.

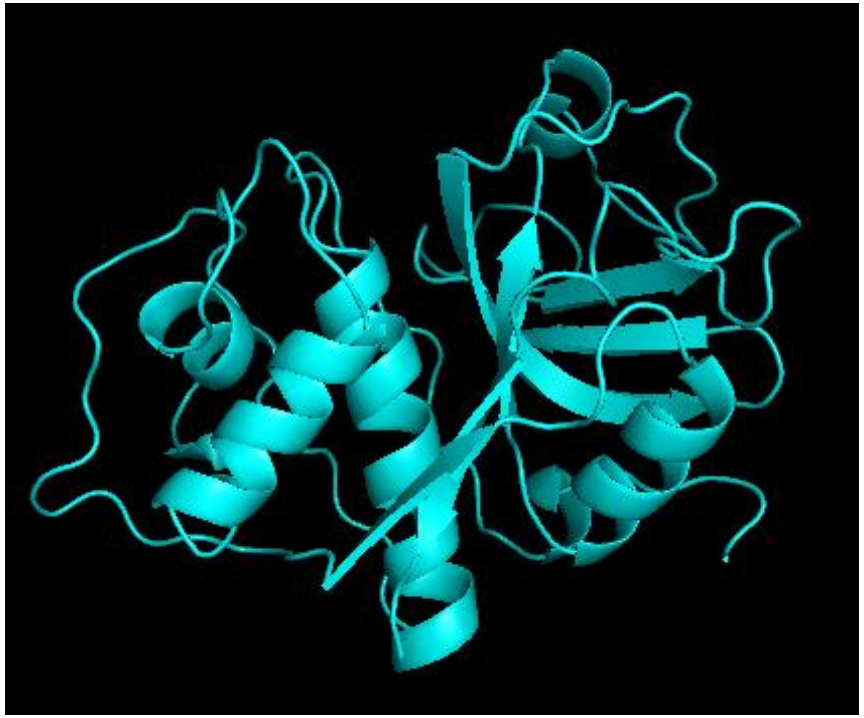

Fig. 1: SWISS-MODEL analysis result: constructed 3D structural model of bromelain viewed using PyMOL

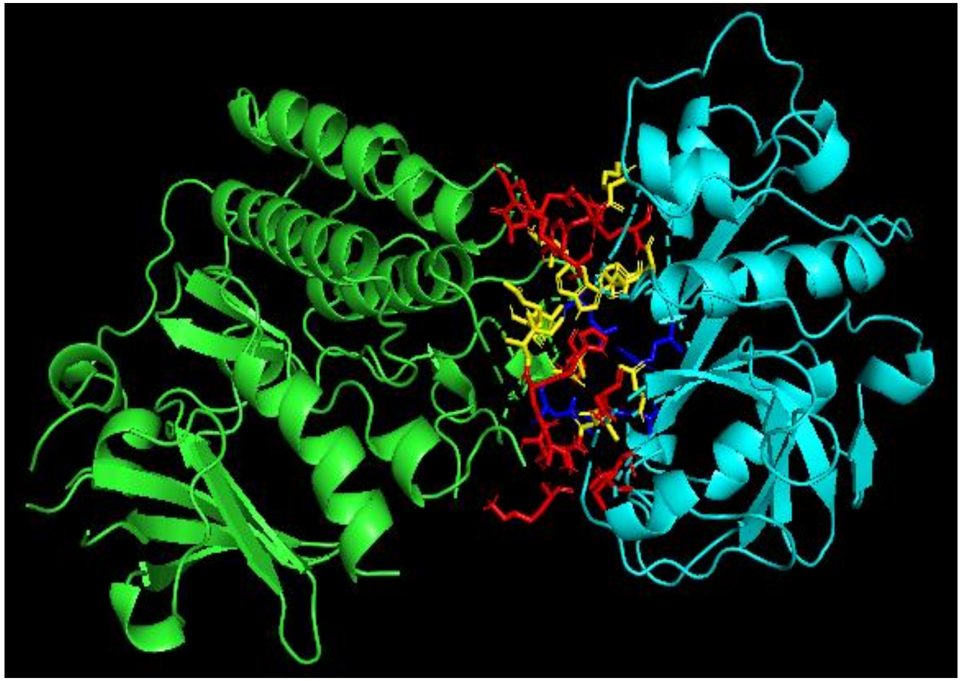

Fig. 2A

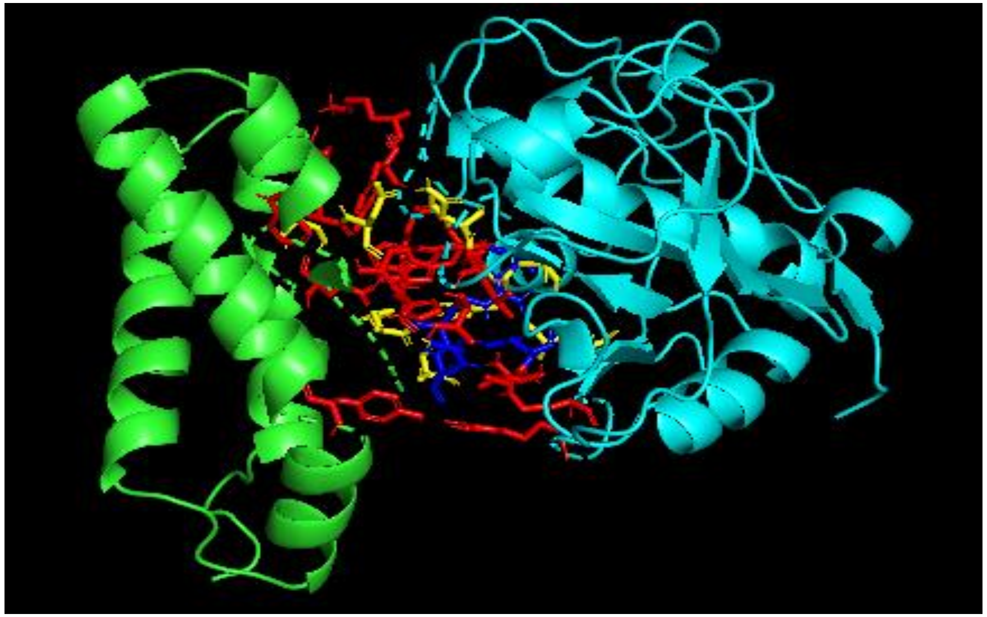

Fig. 2B

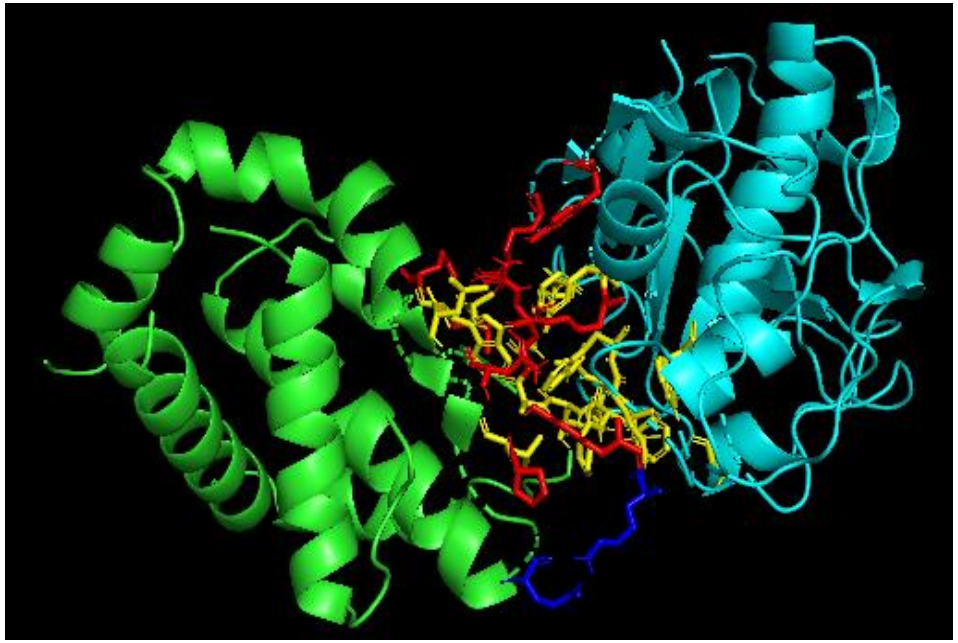

Fig. 2C

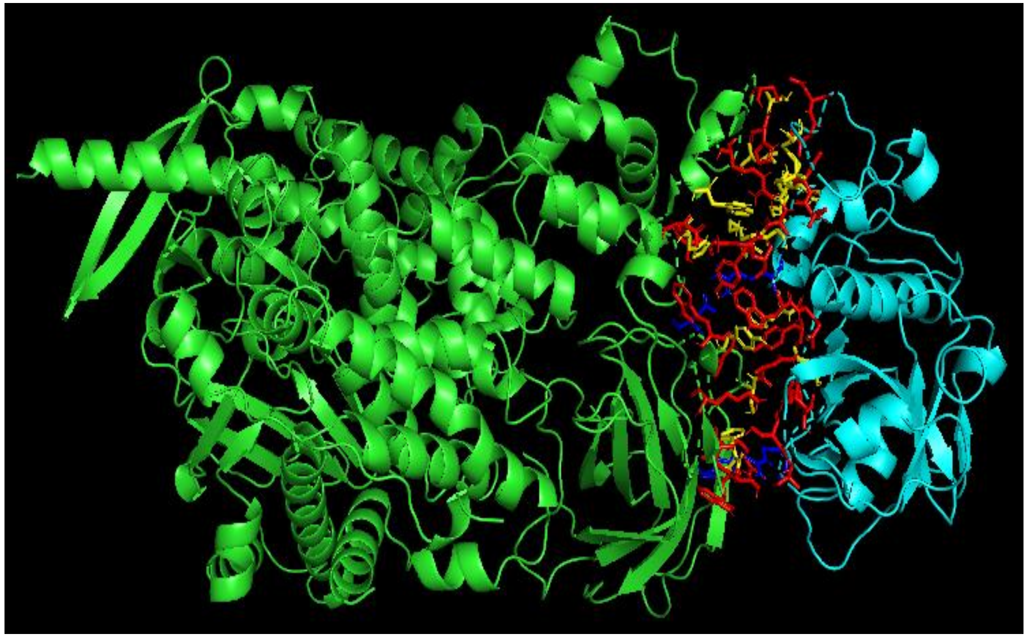

Fig. 2D

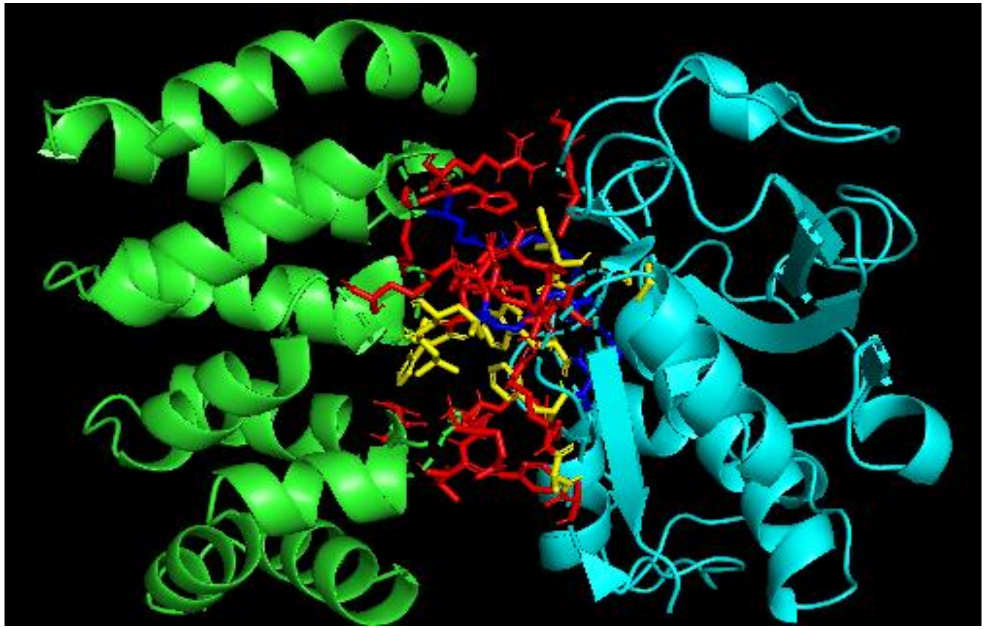

Fig. 2E

Fig. 2 (A, B, C, D and E): PyMOL illustrations of docked complexes: **A** EGFR-bromelain, **B** mTOR-bromelain, **C** BCL2-bromelain, **D** PIK3CA-bromelain, and **E** β-catenin-bromelain. Blue and green ribbon cartoons represent bromelain and target receptor proteins respectively. Red, yellow and blue sticks of the interacting residues represent H-bonds, hydrophobic interactions and salt bridges respectively.

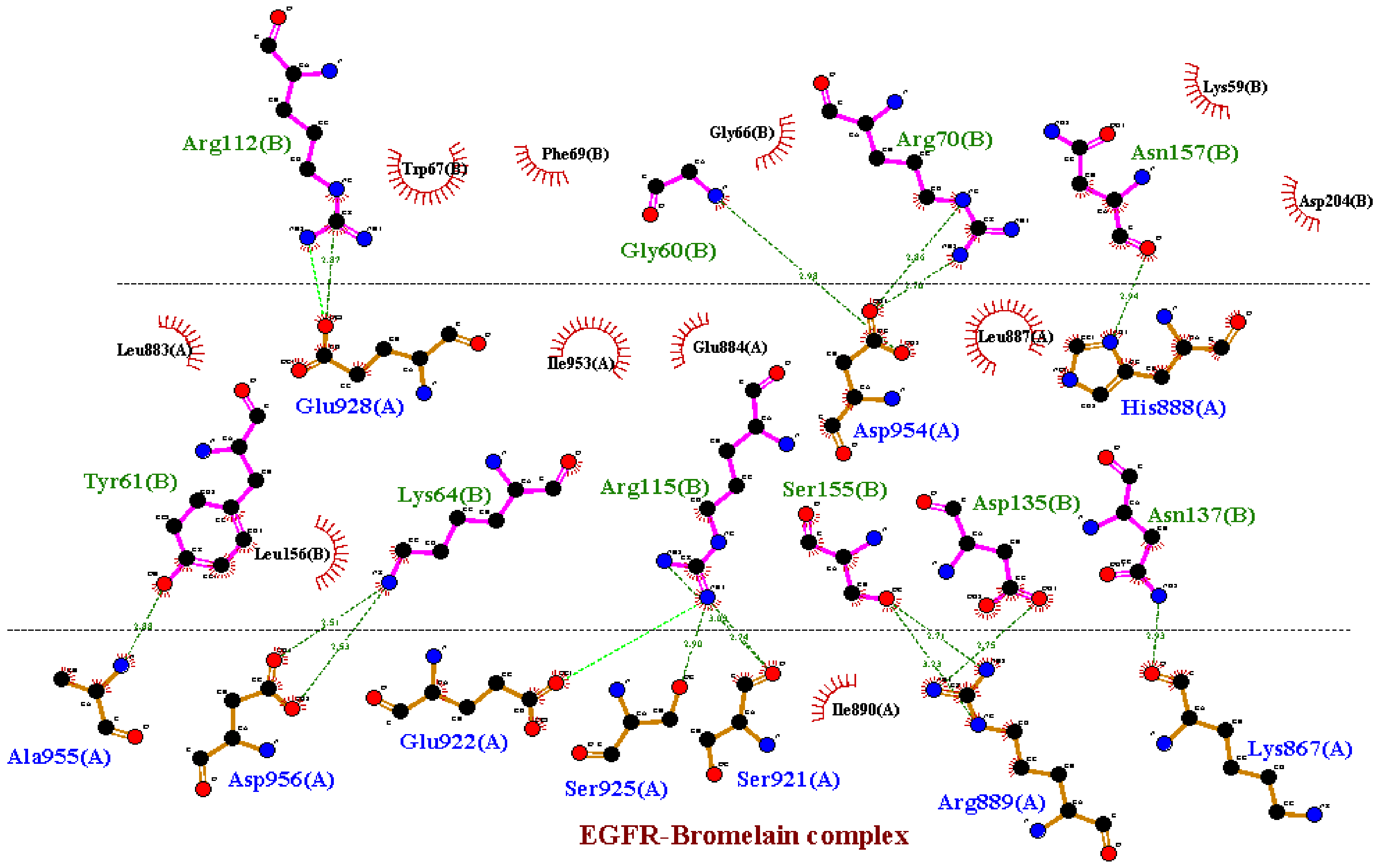
Arg112(B)
Trp67(B)
Phe69(B)
Gly66(B)
Arg70(B)
Lys59(B)
Asn157(B)
Asp204(B)
Gly60(B)
Leu883(A)
Glu928(A)
Ile953(A)
Glu884(A)
Leu887(A)
Asp954(A)
His888(A)
Tyr61(B)
Lys64(B)
Arg115(B)
Ser155(B)
Asp135(B)
Asn137(B)
Leu156(B)
Ile890(A)
Ala955(A)
Asp956(A)
Glu922(A)
Ser925(A)
Ser921(A)
Arg889(A)
Lys867(A)
EGFR-Bromelain complex

Fig. 3A

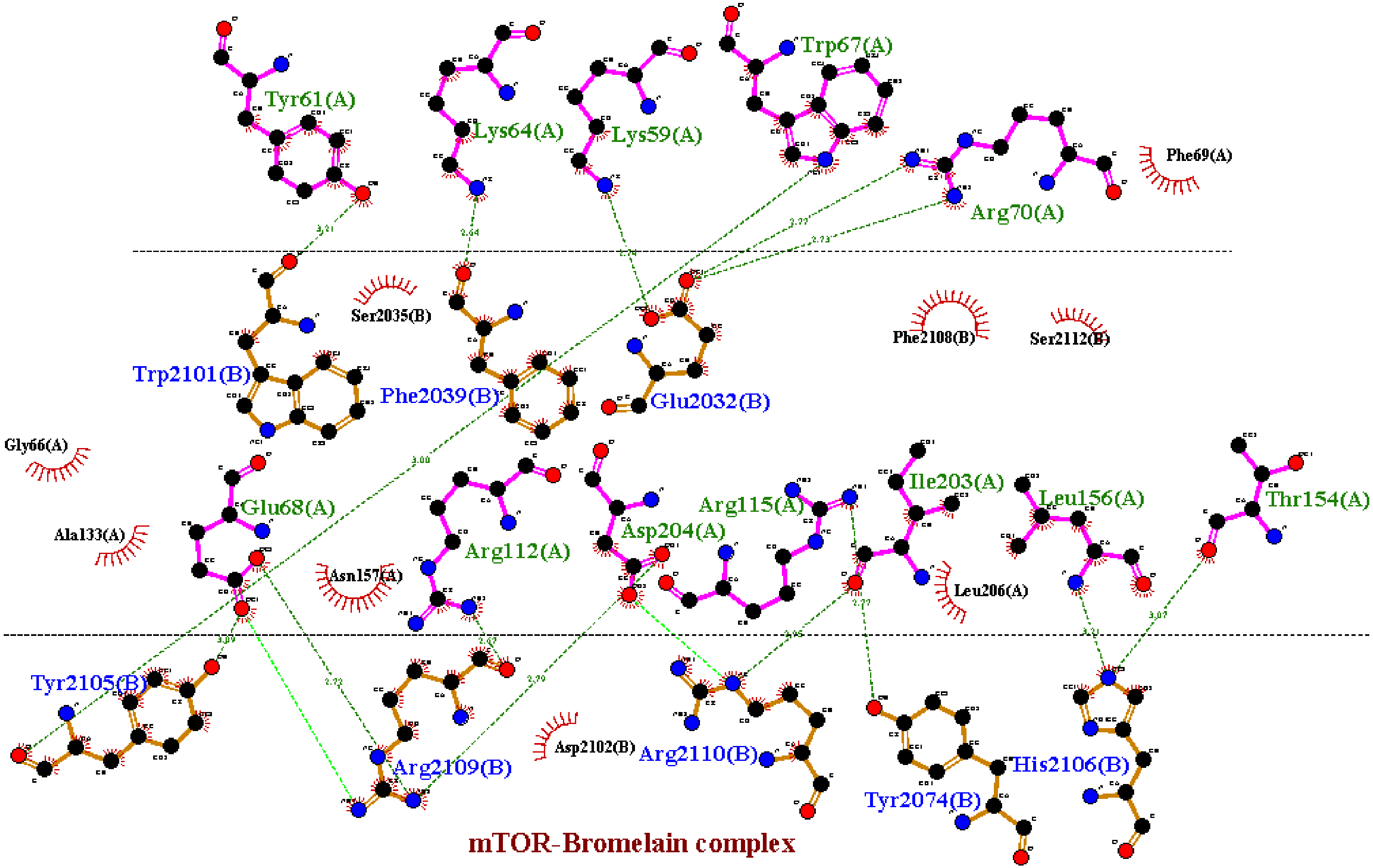

Tyr61(A)
Lys64(A)
Lys59(A)
Trp67(A)
Phe69(A)
Arg70(A)
Ser2035(B)
Phe2108(B)
Ser2112(B)
Trp2101(B)
Phe2039(B)
Glu2032(B)
Gly66(A)
Glu68(A)
Arg112(A)
Asp204(A)
Arg115(A)
Ile203(A)
Leu156(A)
Thr154(A)
Ala133(A)
Asn157(A)
Leu206(A)
Tyr2105(B)
Arg2109(B)
Asp2102(B)
Arg2110(B)
Tyr2074(B)
His2106(B)
mTOR-Bromelain complex

Fig. 3B

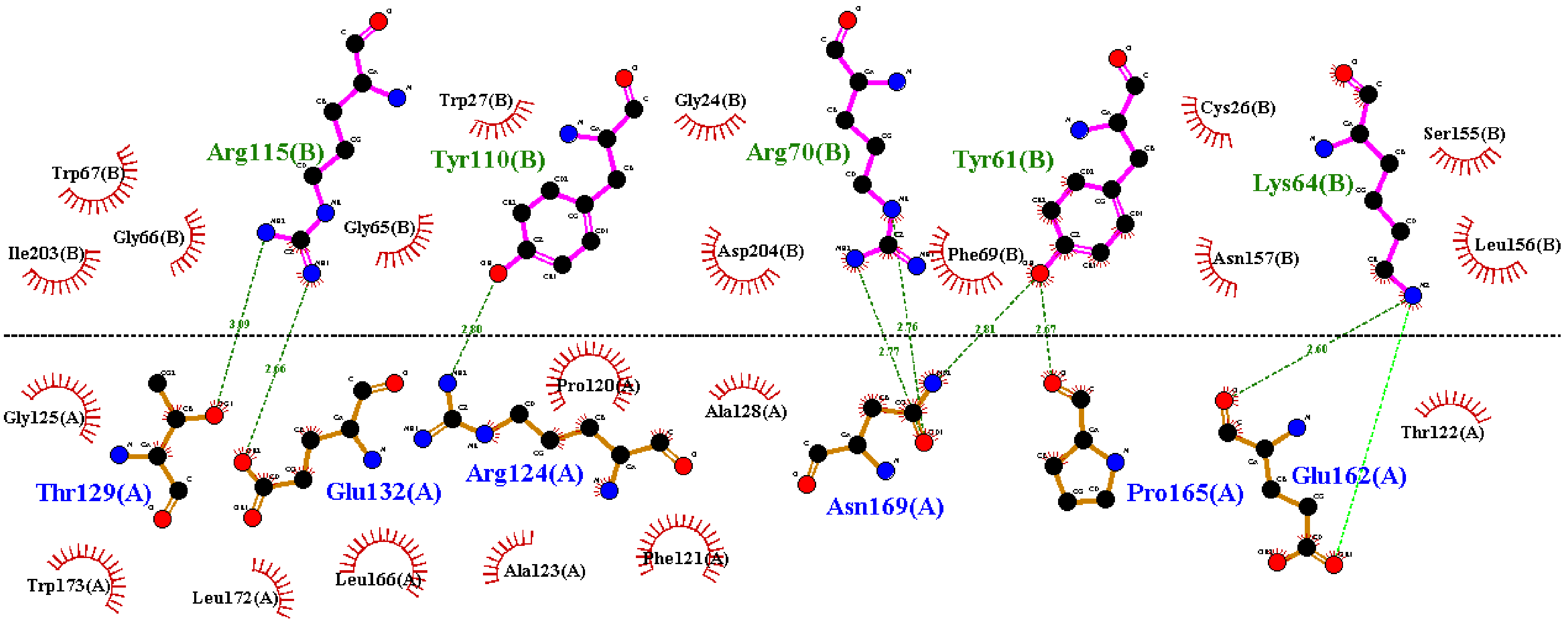

BCL2- Bromelain complex

Fig. 3C

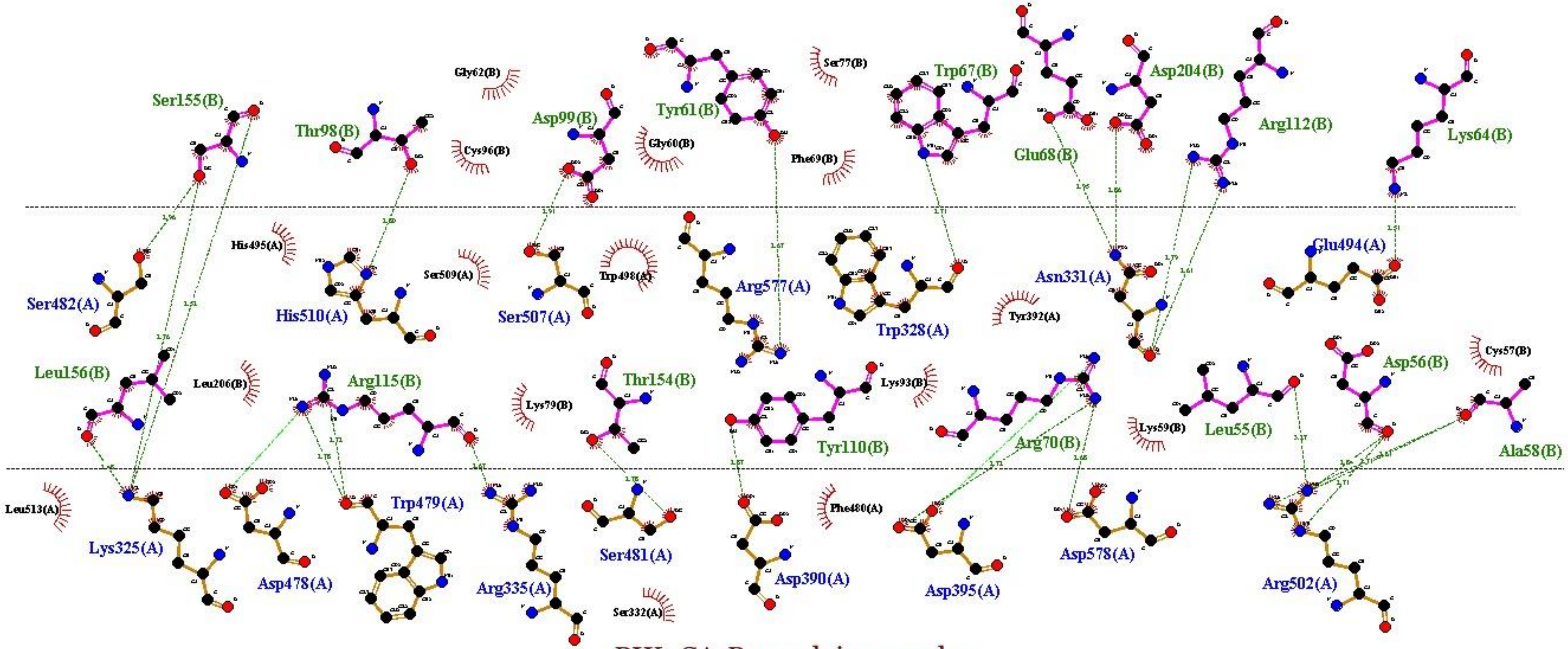

PIK3CA-Bromelain complex

Fig. 3D

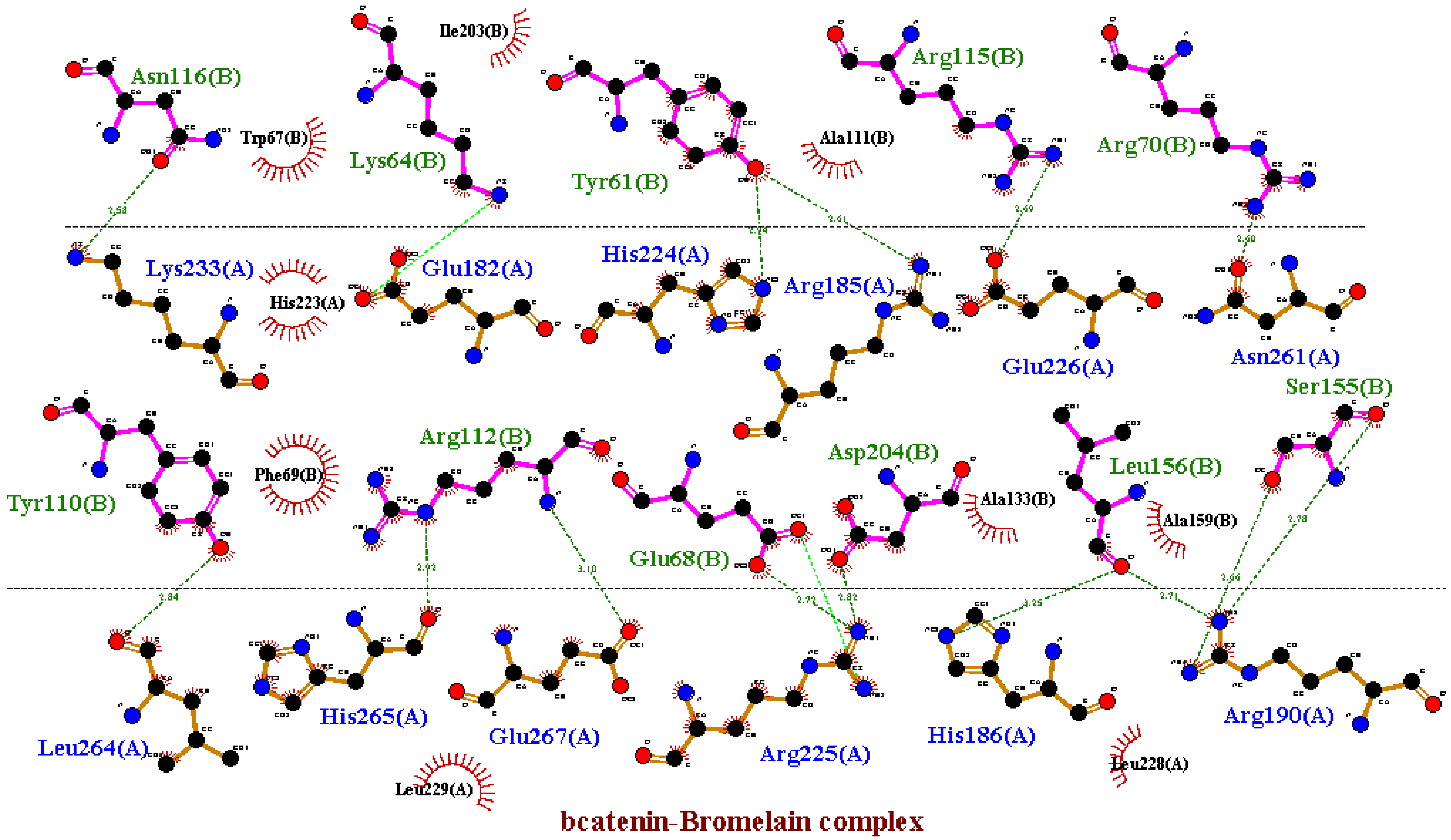
Asn116(B)
Ile203(B)
Trp67(B)
Lys64(B)
Tyr61(B)
Ala111(B)
Arg115(B)
Arg70(B)
Lys233(A)
His223(A)
Glu182(A)
His224(A)
Arg185(A)
Glu226(A)
Asn261(A)
Ser155(B)
Tyr110(B)
Phe69(B)
Arg112(B)
Glu68(B)
Asp204(B)
Ala133(B)
Leu156(B)
Ala159(B)
Leu264(A)
His265(A)
Glu267(A)
Arg225(A)
His186(A)
Arg190(A)
Leu228(A)
Leu229(A)
bcatenin-Bromelain complex

Fig. 3E

Fig. 3 (A, B, C, D and E): LigPlot+ illustrations of docked complexes: **A** EGFR-bromelain, **B** mTOR-bromelain, **C** BCL2-bromelain, **D** PIK3CA-bromelain, and **B** β-catenin-bromelain. Green dotted lines represent hydrogen bonds (H-bonds), neon green lines are the salt bridges, and red-spiked curves indicate hydrophobic residues.
